# Depression symptoms in people with diabetes attending outpatient podiatry clinics for the treatment of foot ulcers

**DOI:** 10.1186/s13047-014-0047-4

**Published:** 2014-11-25

**Authors:** Sue Pearson, Toni Nash, Vanessa Ireland

**Affiliations:** University of Tasmania, School of Medicine, Private Bag 34, Hobart, TAS 7001 Tasmania Australia; Southern Tasmania Area Health Service (STAH)-Podiatry, Royal Hobart Hospital, Hobart, Tasmania Australia

**Keywords:** Diabetes self-management, Depressive symptoms, Foot ulcers, PHQ-9, Antidepressants, Quality of life

## Abstract

**Background:**

The purpose of this study was to examine the prevalence of depressive symptoms, diabetes self-management, and quality of life in people with diabetes and foot ulcers. Ulcer status, mortality and amputations were also assessed at six months follow-up.

**Methods:**

This was a cross-sectional survey of people attending outpatient podiatry clinics at a major tertiary referral hospital. Depressive symptoms were measured using the Patient Health Questionnaire (PHQ). Diabetes self-care was assessed using the Summary of Diabetes Self Care Activities (SDSCA) measure. Health-related quality of life was measured using the physical component summary score (PCS) and the mental component summary score (MCS) of the SF-12.

**Results:**

Of the 60 participants in the study 14 (23.3%) reported mild symptoms of depression (PHQ score 5–9) and 17 (28.3%) moderate to severe depressive symptoms (PHQ score > 9). Twenty-one (35%) met the criteria for previously recognized depression (on antidepressants and/or a diagnosis of depression in the last 12 months) and 17 (28.3%) for depression not previously recognized (PHQ > 4). Seventeen (28%) participants had been receiving antidepressant treatment for a median duration of 104 weeks (IQR 20, 494 weeks). Despite antidepressant treatment 12 participants (70.6% of those taking antidepressants) still reported moderate to severe depressive symptoms at the time of the study. Patients with PHQ scores > 4 reported poorer adherence to diabetes self-care activities including general diet, exercise, blood sugar monitoring and foot care when compared to those participants with PHQ scores < 5. No association was found between physical functioning (PCS) and depressive symptoms. Decreasing mental wellbeing (MCS) was associated with increasing depressive symptoms. At six months follow-up, there were three deaths and three amputations in participants with PHQ scores > 4 compared with no deaths and 2 amputations in participants with PHQ scores < 5. There was no association between depressive symptoms and ulcer healing or ulcer recurrence at the six-month follow-up.

**Conclusions:**

This study found a high prevalence of depressive symptoms both recognized and unrecognized in people with diabetes and foot ulcers. Depressive symptoms were associated with overall poorer diabetes self-management and health-related quality of life (HRQoL). There was no association between depressive symptoms and ulcer outcomes at six-months follow-up.

## Background

Diabetic foot ulcers are one of the most common and costly complications of diabetes occurring in between 15 and 25% of people with diabetes [1]. They are associated with considerable deterioration in quality of life and physical disability [2]. Only two-thirds of ulcers on average will heal within a median time of six months and recurrence of foot ulcers within twelve months is common, occurring in approximately 60% of people [3]. Amputations are also common following deterioration of the ulcer to severe infection or gangrene. Mortality following amputation is high ranging from 39 to 80% at 5 years [4]. Furthermore, once amputated, within 3 years 30-50% of these people undergo amputation of the contralateral-leg [5]. These figures highlight what a serious public health problem this is currently and likely to be in the future given the predicted escalating prevalence of diabetes.

While advances in the treatments of wounds and knowledge about risk factors such as duration of diabetes, persistent hyperglycemia and peripheral neuropathy have assisted in the treatment of these patients [6], significantly less attention has been given to addressing the psychosocial risk factors contributing to diabetic complications and more specifically foot ulcers. It is now well established in the literature that there are higher than normal rates of depression in people with diabetes [7] and that comorbid depression contributes to an increased risk of diabetic complications and mortality [8].

Studies specifically examining the impact of comorbid depression on the incidence and progression of foot ulcers have found depression to be associated with delays in healing [3] and a threefold increased risk of mortality within 18 months of presenting with a first foot ulcer [9]. While depression is likely to occur in response to foot ulceration [10] it is also associated with a 2 fold increased risk of developing foot ulcers when compared to people with diabetes and no depression [11].

The significant burden that comorbid depression contributes to people with diabetes is in part due to the fact that depression is only recognized and appropriately treated in fewer than 25% of people with diabetes [12]. This paper presents the results of a study of patients with diabetes attending outpatient podiatry clinics for the treatment of foot ulcers. Its specific aims were to i) examine the prevalence of depression including previously unrecognized depression, and ii) determine the effect of depression on diabetes-self management, health-related quality of life (HRQoL) and ulcer status at six months follow-up.

## Methods

Participants consisted of 60 people with diabetes and one or more foot ulcers being treated at the foot ulcer clinics run by the Department of Podiatry at the Royal Hobart Hospital in Tasmania, Australia. Both men and women aged 18 years and over who had diabetes (type 1 and 2) were eligible. Participants were excluded if they had a physical or mental condition that prevented them from signing the consent form or filling in the questionnaires. Participants were approached while waiting to be seen by the podiatrist at the clinic. Willing participants could complete the questionnaires while they were waiting for their appointment or they could take them home, complete them and return them in a prepaid envelope. This pilot study was approved by the Tasmanian Health and Medical Human Research Ethics Committee (protocol H11941). During the recruitment period from February to August of 2012, 146 people had scheduled outpatient appointments. Of these patients 75 were excluded and deemed ineligible due to the following reasons; being non-diabetic, having an existing mental health problem, their ulcer had healed or they were being treated for Charcot’s foot, they failed to show up or were unable to sign the consent form. Five refused and 11 failed to return their questionnaires leaving 60 participants in the final analysis of the study.

### Measures

Information from the clinical records included diabetes type, duration, diabetes-related complications, comorbidities, medications including the use of antidepressants. Information about the HbA1C was obtained from the medical records but due to missing data was only available for 41 participants. Data on ulcers was also collected from the patients records. As some patients had more than one ulcer the largest ulcer was classified as the primary ulcer. Severity of the ulcer was classified according to the Texas wound classification scheme and is based on depth, presence of infection and ischemia [13].

Depressive symptoms were assessed using the 9-item Patient Health Questionnaire (PHQ-9). The PHQ is a self-report measure that provides both a diagnosis of major depressive syndrome and a continuous severity score, and is based on the American Psychiatric Association’s DSM-IV criteria for depressive episodes. Participants rate how often in the previous two weeks they have experienced depressive feelings or thoughts. The scale ranges from 0 (not at all) to 3 (nearly every day). Total scores range from 0–27. Validation studies have shown excellent agreement between the self-report PHQ and a clinician-structured interview in general medical outpatients and among people with diabetes. Scores greater than 7 have a sensitivty at 91.9% and specificity of 59.4% [14]. Participants were classified as having mild depressive symptoms if they had a PHQ-9 score from 5–9 and moderate to severe depressive symptoms in the those who scored greater than 9 on the PHQ. Patients categorized as having previously recognized depression included; all participants who were currently on antidepressants for depression and/or those who answered yes to the following question, “over the past 12 months have you been diagnosed by a doctor or other health care professional with depression”. Unrecognized depression included those participants who did not meet the criteria for recognized depression but had a PHQ score greater than 4 (inclusive of mild, moderate and severe symptoms).

Diabetes self-care was assessed using the Summary of Diabetes Self Care Activities (SDSCA) measure. Particpants were required to indicate on how many of the last seven days they attended to self care activities in the areas of general diet, specific diet (fat intake), exercise, blood glucose testing and foot care. Scores range from 0–7 with higher scores indicative of more attention to self management activities. This questionnaire has been shown to be a valid and reliable measure of diabetes self-management in multiple trials with good internal consistency, (mean correlation = 0.47) and acceptable validity (mean correlation = 0.23) [15].

The Medical Outcome Study Short-Form-12 (SF-12) was used to assess HRQoL. It measures physical and mental health by means of two summary scores: a physical component summary (PCS) and mental component summary (MCS) [16]. Both scores range between 0 and 100, with a higher score indicating better health. The SF-12 shows acceptable validity in predicting overall quality of life in people with foot ulcers in terms of physical functioning (r^2^ = 0.26) and mental functioning (r^2^ = 0.372) [17].

### Statistical analysis

Basic despriptive statistics are presented including percentages, means and standard deviations. Group differences were examined using chi-square tests for categorical variables. Fishers exact test was applied where 20% of cell frequencies fell below five. Independent samples *t*-tests and one-way analysis of variance were used for comparison of continuous normally distributed variables and the Kruskal-Wallis test for continuous non-normally distributed variables.

## Results

Table 1 shows the characteristics of participants by PHQ category. The majority of participants were men, retired and receiving a pension, had three or more diabetes related complications in addition to the comorbidities of hypertension and hyperlipidemia. The majority of primary ulcers were superficial and neuroischaemic. Nine (15%) participants had an HbA1c within the recommended guidelines. Of the 60 participants in the study 31 (51.7%) reported having depressive symptoms (PHQ > 4). Of those 31 participants, 14 (23.3% of total sample) could be further classified and having mild depression (PHQ 5–9) and 17 (28.3%) as moderate to severe depression (PHQ > 9). There were no significant differences between the groups except with current use of antidepressants. Seventeen (28.3%) participants were currently taking antidepressants for depression and a greater proportion of them reported moderate to severe depressive symptoms.

**Table 1:** **Characteristics of participants by depression status, as determined by the PHQ**

**Participant characteristics**	**No depressive symptoms PHQ < 5**	**Mild depressive symptoms (PHQ 5–9)**	**Moderate to severe depressive symptoms (PHQ > 9)**	**P value**
N (%)	29 (48.3)	14 (23.3)	17 (28.3)	
Men, n (%)	23 (79.3)	9 (64.3)	13 (76.5)	0.56
Age, years, mean (SD)	65.4 (13.9)	62.4 (13.7)	60.7 (14.4)	0.53
Married or in a relationship, n (%)	18 (62.1)	5 (35.7)	12 (70.6)	0.13
Live alone, n (%)	7 (25.9)	4 (33.3)	1 (9.1)	0.37
High school education or less, n (%)	18 (62.1)	9 (64.3)	10 (58.8)	0.95
Pension as main source of income, n (%)	22 (75.9)	11 (78.6)	12 (70.6)	0.79
Diabetes type 2, n (%)	23 (79.3)	13 (92.9)	12 (70.6)	0.30
*Diabetes duration*, n (%)				
>10 years	18 (62.1)	11 (78.6)	12 (70.6)	0.54
Insulin, n (%)	20 (69)	12 (85.7)	11 (64.7)	0.30
*Diabetes complications, n (%)*				
1	6 (20.7)	4 (28.6)	3 (17.6)	0.96
2	9 (31)	4 (28.6)	6 (35.3)	
3+	14 (48.3)	6 (42.9)	8 (47.1)	
Hypertension, n (%)	19 (65.5)	11 (78.6)	12 (70.6)	0.68
Hyperlipidemia, n (%)	17 (58.6)	7 (50)	12 (70.6)	0.49
HbA1C level ≤7.00%, n (%)	6 (26.1)	2 (25)	1 (10)	0.57
Smoking	3 (10.3)	1 (7.1)	4 (23.5)	0.35
Taking antidepressants for depression, n (%)	3 (10.3)	2 (14.3)	12 (70.6)	≤0.0001
*Texas Wound Classification, n (%)*				
Superficial wound	22 (75.9)	11 (84.6)	14 (82.4)	0.42
Infected	7 (24.1)	1 (8.3)	3 (17.6)	0.63
Neuroischaemic	27 (93.1)	13 (92.9)	15 (88.2)	0.69
Ulcer duration >6 months, n (%)	25 (86.2)	9 (64.3)	14 (82.4)	0.23

Due to missing data for A1C: no depression n = 23 and depression n = 18.

Complications: retinopathy, renal impairment, stroke, heart disease, peripheral arterial disease, peripheral neuropathy.

Of the seventeen participants who were on antidepressants for depression there was available data on the duration of antidepressant treatment for thirteen. The duration of antidepressant treatment was a median of 104 weeks (IQR 20 and 494 weeks). Of these participants 6 (46.2%) had been on antidepressants for more than two years and 3 (23.1%) for more than 10 years.

Table 2 shows mean scores on the SDSCA and the SF-12 by PHQ category. This SDSCA data was skewed and therefore analyzed using nonparametric methods. Participants with no depressive symptoms (PHQ < 5) reported higher scores on all domains of the SDSCA except specific diet. Significant differences between the groups on specific diet occurred between participants with PHQ scores < 5 (no depressive symptoms) and those with mild depressive symptoms PHQ 5–9), p = 0.021 and between those with mild depressive symptoms and moderate to severe symptoms (PHQ > 9), p = 0.036. Differences between the groups on general diet were approaching significance (p = 0.06), all other comparisons between the three groups were not significant. Scores on the SF12 were normally distributed. Scores on physical functioning (PCS) were not significantly different between the groups. This analysis was adjusted for age as this was strongly associated with measures of physical functioning (r = −0.370, p = 0.004). There was a significant difference between the groups in mental functioning (MCS) following adjustment for age also (r = 0.374, p = 0.004). Post-hoc comparisons using the Sheffe test found these differences to be significant between participants with PHQ scores < 5 (no depressive symptoms) and those with mild depressive symptoms PHQ 5–9), p = 0.001 and between those with mild depressive symptoms and moderate to severe symptoms (PHQ > 9), p = 0.001.

**Table 2:** **Diabetes self management and quality of life in participants according to PHQ score**

	**No depressive symptoms PHQ < 5**	**Mild depressive symptoms (PHQ 5–9)**	**Moderate to severe depressive symptoms (PHQ > 9)**	**P value**
	**Mean (± sd)**	**Mean (± sd)**	**Mean (± sd)**	
***SDSCA scores***				
General diet	5.9 (1.9)	5.4 (2.2)	4.3 (2.4)	0.06
Specific diet	4.2 (1.3)	5.3 (1.5)	3.8 (2.2)	0.04
Exercise	1.8 (2.1)	0.9 (1.3)	1.1 (2.0)	0.19
Blood sugar monitoring	5.7 (2.4)	4.9 (2.6)	4.2 (2.8)	0.11
Foot care	5.3 (2.0)	5.0 (2.4)	4.3 (2.2)	0.19
***SF-12 scores***				
Physical component score	33.4 (8.3)	33.5 (5.5)	37.4 (6.1)	0.23
Mental component score	53.7 (8.6)	48.3 (8.4)	42.9 (8.3)	≤0.001

*sd* = standard deviation.

Twenty-one participants (35%) met the criteria for prior recognition of depression and 17 (28.3%) for depression not previously recognized. There were no significant differences between these two groups on any demographic or clinical variables. Table 3 shows the break down of depressive symptoms as reported on the PHQ according to these two groups. Significantly more participants with previously unrecognized depression reported depressive symptoms in the mild category compared to people with recognized depression. Whereas a greater proportion of participants with recognized depression reported moderate to severe depressive symptoms compared to those with unrecognized depression. Chi-square analysis showed these groups to be significantly different at P ≤ 0.0001.

**Table 3:** **Depression symptoms on the PHQ in previously recognized and unrecognized depression categories**

**PHQ category**	**Depressive symptoms previously recognized**	**Depressive symptoms not previously recognized**
	**(N = 21)**	**(N = 17)**
	N (%)	N (%)
No depressive symptoms (PHQ ≤ 4)	7 (33.3)	0
Mild depressive symptoms (PHQ 5–9)	2 (9.5)	12 (70.6)
Moderate to severe depressive symptoms (PHQ > 9)	12 (57.1)	5 (29.4)

Table 4 shows the data for the 6-month follow-up of primary ulcer status. Due to the number of categories for comparison and the resulting small number of participants in the cells the categories were collapsed into two categories, comparing participants with a PHQ score <5 (no depressive symptoms) to those with a PHQ score >4 (inclusive of mild, moderate and severe depressive symptoms). Data was missing for six participants, three were lost to follow up and three had died. There were no significant differences between the two groups. The three participants who were deceased at the time of follow-up all had PHQ scores >4. Of the three who had amputations in this group, two were toe amputations and another a below knee amputation. There were two toe amputations in the group with PHQ score <5 and no deaths were recorded in this group.

**Table 4:** **Six-month follow-up of primary wound status by PHQ category**

	**PHQ < 5 (N = 26)**	**PHQ > 4 (n = 28)**	**P value**
*Primary wound status*	N (%)	N (%)	
Healed	13 (50)	16 (57.1)	0.93
Diminished in size	6 (23.1)	5 (17.9)	
Increased in size	3 (11.5)	3 (10.7)	
No change in size	2 (7.7)	1 (3.6)	
Amputation	2 (7.7)	3 (10.7)	
New ulceration at a different site, n (%)	8 (33.3)	6 (24)	0.47

## Discussion

The present study examined the prevalence of depressive symptoms including previously unrecognized symptoms, diabetes self-management, quality of life and ulcer status at six months follow-up in people with diabetes and foot ulceration attending outpatient podiatry clinics. There are a number of important findings from the study. Firstly, there was a high prevalence of depressive symptoms with around half of the participants reporting PHQ scores > 4. Similar findings have been reported in a number of other studies. One study using diagnostic criteria to measure depression found around a third of participants had clinically significant minor or major depression [9]. The second study using the Beck Depression Inventory found moderate depression in 64% of participants and severe depression in 10% [18]. These prevalence’s are generally higher than the prevalence of depression reported in people with diabetes without foot ulcers, which ranges from 11% using standardized diagnostic interviews to 31% when assessed by self-report [19]. Higher prevalence’s in participants with foot ulcers may be explained in part by the increased burden associated with having a foot ulcer.

Secondly, this study also found an association between depressive symptoms and poorer diabetes self-management. Gonzalez and colleagues report findings consistent with this in addition to an association with poorer medication adherence [20]. While there was no association found in this study with physical functioning and depressive symptoms the scores on the SF-12 for this component were generally low (overall mean 34.6, SE 0.9) compared with age and sex matched data for the Australian population with diabetes (mean 44.0, SE 1.0) [21]. Goodridge and colleagues have previously reported similar low scores on the SF-12 for physical functioning when comparing groups of participants with healed and unhealed ulcers [22]. Caution should be applied when interpreting the result from this study due to the generic nature of the SF-12 measure. The use of a foot-ulcer specific measure such as the NeuroQoL may have been better at detecting an association between the presence of foot ulcers and poorer quality of life. Validation studies comparing the NeuroQoL with the SF-12 have shown the NeuroQoL to be a superior instrument in this regard [17]. In the absence of having used a disease-specific measure in this study it is difficult to know with confidence the results from the SF-12 are directly related to the presence of a foot ulcer when there are potentially other conditions that might also explain the low physical functioning scores. Decreasing mental wellbeing (MCS) on the SF-12 was associated with increasing depressive symptoms. This is not so surprising as the MCS and PHQ both measure constructs of mental well-being. They are, however different measures. The MCS is a more general assessment of emotional problems and their impact on work, daily activities and social activities over the last 4 weeks whereas the PHQ-9 is a more focused measure of depressive symptoms. The MCS provides some additional information over and above that measured by the PHQ regarding the influence of poor mental health on other domains of life.

Thirdly, this study found no association between depression and ulcer outcomes at six-months follow-up. There have been very few studies including this one to date and the results have been inconsistent. Monami and colleagues [3] found significant associations between impaired ulcer healing at six months and recurrence of ulcers at twelve months and greater depressive symptoms. In contrast, a study by Winkley and colleagues [2] using a larger cohort and a longer follow-up period found no association between greater depressive symptoms and ulcer healing at eighteen months. This study by Winkley et al. did however report a significant association with increased mortality at eighteen months and in a subsequent five year follow-up of the cohort [23]. While this is consistent with an increase in mortality found in our study in those with depressive symptoms versus those without we did not test this statistically due to the small sample size. We also found no differences in amputations between the groups in association with depression. This is in contrast to a previous study by Williams and colleagues [24] who found an increase of 33% in amputation risk associated with diagnosed depression over a four year period. The large sample size of over 600,000 participants in this study and extended follow-up are probably explanations for the discrepancies in findings.

Another important finding from this study was that 28% of participants had previously unrecognized depression, which is also consistent with previous studies [12,25,26]. Unrecognized depression in this study was associated with a higher proportion of participants having mild depression. A possible and perhaps obvious explanation for this is that moderate to severe symptoms of depression are more apparent and therefore easier to diagnose. These patients may also be more likely to seek treatment. Identification of depression in people with diabetes can be problematic as some of the symptoms of depression are also symptomatic of a diagnosis of diabetes and may explain why it goes unrecognized.

The large majority of participants with previously recognized depression were being treated with antidepressants and most of them for more than two years. Maintenance pharmacotherapy is sometimes a recommended treatment option in cases where there are high rates of relapse. Certainly depression in people with diabetes does tend to be more chronic and long lasting [27]. In this study we have shown, however, that such maintenance pharmacotherapy was not effective in treating their depression as many of these people continued to experience moderate to severe depression as indicated by their scores on the PHQ. A concern of long-term treatment with some antidepressants particularly with regard to people with diabetes is the side effect of weight gain [28] and potential to delay wound healing [29].

An important question that this study raises is whether or not patients who had been on long-term antidepressants had received any other form of psychotherapeutic interventions for their depression. The benefits of psychotherapeutic interventions including cognitive behavioral therapy are well documented [30]. This meta-analysis of fourteen randomized trials found the most significant effects on depression and glycemic control in people with diabetes was associated with psychotherapeutic interventions when compared with pharmacological interventions only or a combination of both pharmacological and psychotherapeutic interventions. Major policy changes introduced in Australia in 2006 to increase access to mental health services have shown that around 46% of people with mental health problems accessed specialist services in 2009–2010 a significant improvement from 37% in 2006. However, what is not known is whether people accessing these services received evidence-based therapies and what their outcomes were [31]. This in addition to other studies acknowledging a treatment gap regarding evidence-based treatment of mental health problems leads one to speculate that the patients in this study may not have received these other forms of non-pharmacological interventions [32-35].

For a number of years international diabetes guidelines have been recommending routine screening of patients with diabetes for depression and diabetes-related distress [36,37]. Guidelines recently released in Australia by the Royal Australasian College of General Practitioners also acknowledge the need for addressing the psychological well-being of these patients [38]. This rationale is based on substantial evidence of an increased prevalence of emotional problems in people with diabetes [7], its association with adverse outcomes including diabetes-related complications [23,39] and the availability of effective treatments [30]. This equates to a strong argument that such problems should be addressed and that these individuals need to be identified. Whether the implementation of routine screening in secondary care is the most efficient and cost-effective way of doing this remains controversial. A recent study [40] evaluating routine screening in an outpatient diabetes clinic found up to 30% of patients were missed by screening and only a small number of patients who screened positive were happy to be referred on for further treatment. Those missed by screening were more likely to be smokers and younger, have high HbA1c, show lower adherence to diabetes care in general, and therefore also be more likely to be at a greater risk of having depression [41]. A problem with the screening debate is a lack of empirical evidence in terms of rigorous randomized controlled trials around screening. Issues that need to be addressed include feasibility and cost-effectiveness, where screening should take place (primary or secondary care), the best way to identify ‘high-risk patients’ what resources are required and what constitutes a successful outcome for the patients (clinical endpoints, quality of life, reductions or delay in complications). What is not controversial is a general recognition by health care professionals that issues around psychological distress and depression in these patients deserve attention in the clinical setting.

Limitations of the study include the small sample size and that housebound and community clinic patients with ulcers would not have been identified. This limits the generalization of the study results to some degree and the statistical analysis. An additional limitation was the missing data for HbA1C. It was not in the protocol to measure this at the time of consent and was reliant on existing documentation in the medical records. It raises questions about the representativeness of the data. Also there are inherent limitations with self-report questionnaires such as under or over reporting.

## Conclusion

In conclusion this study found a high prevalence of depression as determined using the PHQ in people with diabetes and foot ulcers. Most with severe depression were being treated with antidepressants, however the prolonged use of antidepressants appears to be ineffective in the majority of cases. There was also a high prevalence of participants with mild to moderate depressive symptoms that were not previously identified. This is concerning given the evidence from other research in this area of an association of mild depression in people with diabetes and foot ulcers with increased mortality [23]. It has been recognised that mild depression in people with diabetes is a significant predictor of severe depression at two years follow-up [42]. With the increasing incidence of diabetes globally, identification of previously unrecognized depression (mild and moderate to severe) in people with diabetes either before the onset of complications or those with existing complications is important as it provides the opportunity for early intervention. From a public health perspective the provision of evidence-based therapies for those with depression and foot ulceration should form part of the holistic management of this group with complex medical and psychosocial needs.

## Abbreviations

PHQ: Patient Health Questionnaire
SDSCA: Diabetes self-care was assessed using the Summary of Diabetes Self Care Activities
PCS: Physical Component Summary score
MCS: Mental component summary score
